# Retraction: Mechanisms of M2 Macrophage-Derived Exosomal Long Non-coding RNA PVT1 in Regulating Th17 Cell Response in Experimental Autoimmune Encephalomyelitis

**DOI:** 10.3389/fimmu.2021.773900

**Published:** 2021-09-24

**Authors:** Frontiers Editorial Office

**Affiliations:** Frontiers Media SA, Lausanne, Switzerland

The journal retracts the 04 September 2020 article cited above.

Following publication, concerns were raised regarding the integrity of the data in the published figures. The authors failed to provide a satisfactory explanation during the investigation, which was conducted in accordance with Frontiers’ policies.

This retraction was approved by the Chief Editors of Frontiers in Immunology and the Chief Executive Editor of Frontiers. The authors agree to this retraction.

